# BUILDING CAPACITY TO CARE FOR OLDER PEOPLE! HOW IS CARE OF THE OLDER PERSON TAUGHT IN AUSTRALIAN SCHOOLS OF NURSING?

**DOI:** 10.1093/geroni/igz038.007

**Published:** 2019-11-08

**Authors:** Deirdre M Fetherstonhaugh, Jo-anne Rayner, Elizabeth Beattie, Ann Harrington, Yun-Hee Jeon, Wendy Moyle, Deborah Parker, Chris Toye

**Affiliations:** 1 La Trobe University, Victoria, Australia; 2 Queensland University of Technology, Queensland, Australia; 3 Flinders University, South Australia, Australia; 4 Sydney University, New South Wales, Australia; 5 Griffith University, Queensland, Australia; 6 University of Technology, New South Wales, Australia; 7 Curtin University, Western Australia, Australia

## Abstract

As the Australian population ages the demand for nursing care which focuses on responding to the needs of the older person will increase. Few newly graduated Registered Nurses (RNs) currently enter the aged care workforce and few select a career in caring for older people; yet older people are the largest patient group in most health care environments. This research, conducted by the Australian Hartford Consortium of Gerontological Nursing Excellence (Aus-HCGNE), explored how care of the older person is currently taught in Australian schools of nursing (SoN). The interview guide included questions about: whether care of the older person is taught in separate subjects or integrated across the curriculum; academics’ qualifications; subject content; and aged care clinical placements. The head of each of the 33 Australian schools of nursing was contacted, invited to participate and asked to nominate the appropriate academics (undergraduate/curriculum co-ordinators) who would be the most appropriate person to participate in the interview. These academics were then contacted, written informed consent was obtained, interviews were scheduled and completed. This research is timely given the current Royal Commission into Aged Care Quality and Safety in Australia, one focus of which is nurses in residential aged care in respect to numbers, education and competence. This research will be completed by mid-2019. The results will be fed back to SoN to inform the development of their curricula and the preparation of future RNs who will undoubtably need to be expert in the care of older people across the health sector.

